# Dynamics of predator-prey habitat use and behavioral interactions over diel periods at sub-tropical reefs

**DOI:** 10.1371/journal.pone.0211886

**Published:** 2019-02-06

**Authors:** Fabio Campanella, Peter J. Auster, J. Christopher Taylor, Roldan C. Muñoz

**Affiliations:** 1 Centre for Environment, Fisheries and Aquaculture Science, Lowestoft, United Kingdom; 2 University of Connecticut, Department of Marine Sciences, Groton, Connecticut, United States of America; 3 Mystic Aquarium, Mystic, Connecticut, United States of America; 4 National Oceanic and Atmospheric Administration, National Centers for Coastal Ocean Science, Beaufort Laboratory, Beaufort, North Carolina, United States of America; 5 National Oceanic and Atmospheric Administration, National Marine Fisheries Service, Southeast Fisheries Science Center, Beaufort Laboratory, Beaufort, North Carolina, United States of America; Tanzania Fisheries Research Institute, UNITED REPUBLIC OF TANZANIA

## Abstract

The dynamics of fish communities at tropical and sub-tropical rocky reefs are influenced in many cases by predation activity and predator-prey interactions. These processes usually follow specific diel patterns in reef areas with higher rates of these interactions occurring during the crepuscular periods. However, other factors such as habitat complexity and species-specific behavior may alter these patterns, increasing variability in species interactions. A better understanding of the dynamics of these patterns and processes would allow us to manage and monitor fish communities in these productive and vulnerable areas more efficiently. We investigated behavioral changes of predators and prey fish in sub-tropical “live-bottom” (sandstone) reefs at Gray’s Reef National Marine Sanctuary (GRNMS), located 20 nautical miles off the coast of Georgia, USA, using fisheries acoustic methods in association with visual census and direct observation using SCUBA. Changes in co-location and habitat preferences of predators and prey over time throughout the diel cycle were investigated using species distribution models (MAXENT) based on habitat predictors and by means of spatial statistics. The results indicate that predator and prey distribution patterns changed considerably throughout the day. Prey and predator species exhibited complex spatial dynamics and behavior over diel periods, with prey modifying patterns of habitat use and spatial distribution, likely as a response of their interactions with predators. Crepuscular periods were confirmed to be the most active phases in terms of predator-prey interactions and consequently the most variable. The combination of tools and approaches used in this study provided valuable sources of information that support the inferences of predation risk-driven habitat selection of prey in this sub-tropical reef system.

## Introduction

Higher trophic level predators can have profound effects on the structure of both terrestrial and marine communities [1]. Examples of predators driving fish community structure are perhaps best known from tropical and sub-tropical reefs. These areas are regulated in many cases by piscivorous fish that exert top-down control over intermediate and lower trophic levels [2,3,4,5].

Predators can have a direct effect on prey (density mediated) by increasing their mortality rate and consequently reducing population size. Moreover, non-lethal effects (trait mediated) can also occur, which include modification of behavior (e.g., shifts in feeding and shelter habitats) and traits such as growth rate, fitness, spawning and recruitment success [6,7,8,9,10]. These ecological effects are often a result of behaviors driven by the perception of predation risk by the prey that can vary spatially and temporally [11].

One of the primary mechanisms used by prey as a response to higher levels of predation threat is modifying their patterns of habitat use, moving toward areas with lower per-capita predation risk [12]. The shift in habitat use between high-risk and reduced-risk environments within the range of potential prey habitats (the aptly named landscape of fear [13]) has been observed in many ecological settings [14,15,16]. Changes in the habitat use of prey induced by high densities of predators generally result in a reduction of prey foraging efficiency. This could result in a decrease in the per capita growth and an increase of the time that they are more susceptible to predators [17]. Following the optimal foraging concept, the response to a predation threat requires an energetic trade-off between trophic efficiency and avoiding predators to avoid predation [18,19].

Spatial heterogeneity of the seascape could have strong effects on predator behaviors and prey responses. Habitat with high structural and environmental complexity can reduce encounter rates between predators and prey by providing refugia from detection and attack. At the same time areas with high productivity could decrease density related competition between prey species. Indeed, the relationship between habitat complexity and predator-prey interactions is not always linear and can be regulated by several factors such as prey density [20], shelter characteristics, prey size, behavioral characteristics of predators and prey (pelagic vs demersal, resident vs transient, schooling vs individual), inter-specific relationships, and anthropogenic interactions [21,22].

Most recent studies on behavior of predators have been focused on the functional details of predator-prey interactions (e.g., [23,24]) but the dynamics of predation activity vary throughout diel periods [25,26]. Generally, the diel pattern of fish behavior is divided in two phases, one that is focused on food seeking and a resting phase where individuals avoid predators. There is significant variation in the timing of these phases depending on the species’ trophic role, and the environmental conditions. For example, in tropical and subtropical reefs many piscivorous species exhibit a clear diel pattern. During the daytime, resident demersal piscivorous species (e.g. gag grouper—*Mycteroperca microlepsis*, red snapper–*Lutjanus campechanus*) exhibit a cryptic behavior and are actively feeding. Transient predators (e.g. barracuda–*Sphyraena barracuda*, amberjack—*Seriola* spp) may also feed during the day relying on errors and misjudgment of the prey. The crepuscular period (dawn and dusk) is a transition phase that is often linked to a sharp change in the behavior of many species. During this period, predators can exhibit higher predation activity, taking advantage of the high vulnerability of the prey due to low light levels and the transition from shoaling behavior to individual behavior and vice versa [25]. Few studies have attempted to describe the temporal variability and spatial extent of predator-prey interactions, in particular over diel time scales. A better understanding of this pattern and dynamics would facilitate predictions of fish distribution and density, population and community status, interpretation of monitoring surveys, and assessments of the strength and importance of species interactions for conservation and management [27].

The challenges to studying these systems is the need for synoptic and simultaneous non-obstructive observations of predator and prey coincident over both space and time as well as over the habitat mosaic. Fisheries acoustics has been largely used in marine environments to study fish population dynamics, especially pelagic species, providing high temporal and spatial resolution data that are not easy to obtain using other fishery-independent methods such as diver visual census, trawls, and optical tools such as camera sleds [28]. The use of fisheries acoustics for the study of demersal and semi-demersal species has increased in the recent years [29,30,31]. The combination of fisheries acoustics with visual quantitative and qualitative observations can overcome the limitation of acoustics alone in detecting and discriminating species associated with seafloor features.

Here we report on an investigation of the behavioral changes in predators and prey over time, especially during crepuscular periods, using fisheries acoustics in association with direct observations at sub-tropical live-bottom reefs. Our study took place in Gray’s Reef National Marine Sanctuary off the southeast coast of the United States (NW Atlantic). Moreover, habitat modeling methods were used to link the observed dynamics of habitat selection of predators and prey over the 24-hour period to habitat metrics as response variables, thereby assessing the landscape context for predicting responses of co-occurring predators and prey in similar environmental settings.

## Materials and methods

### Study area

**Fig 1:**
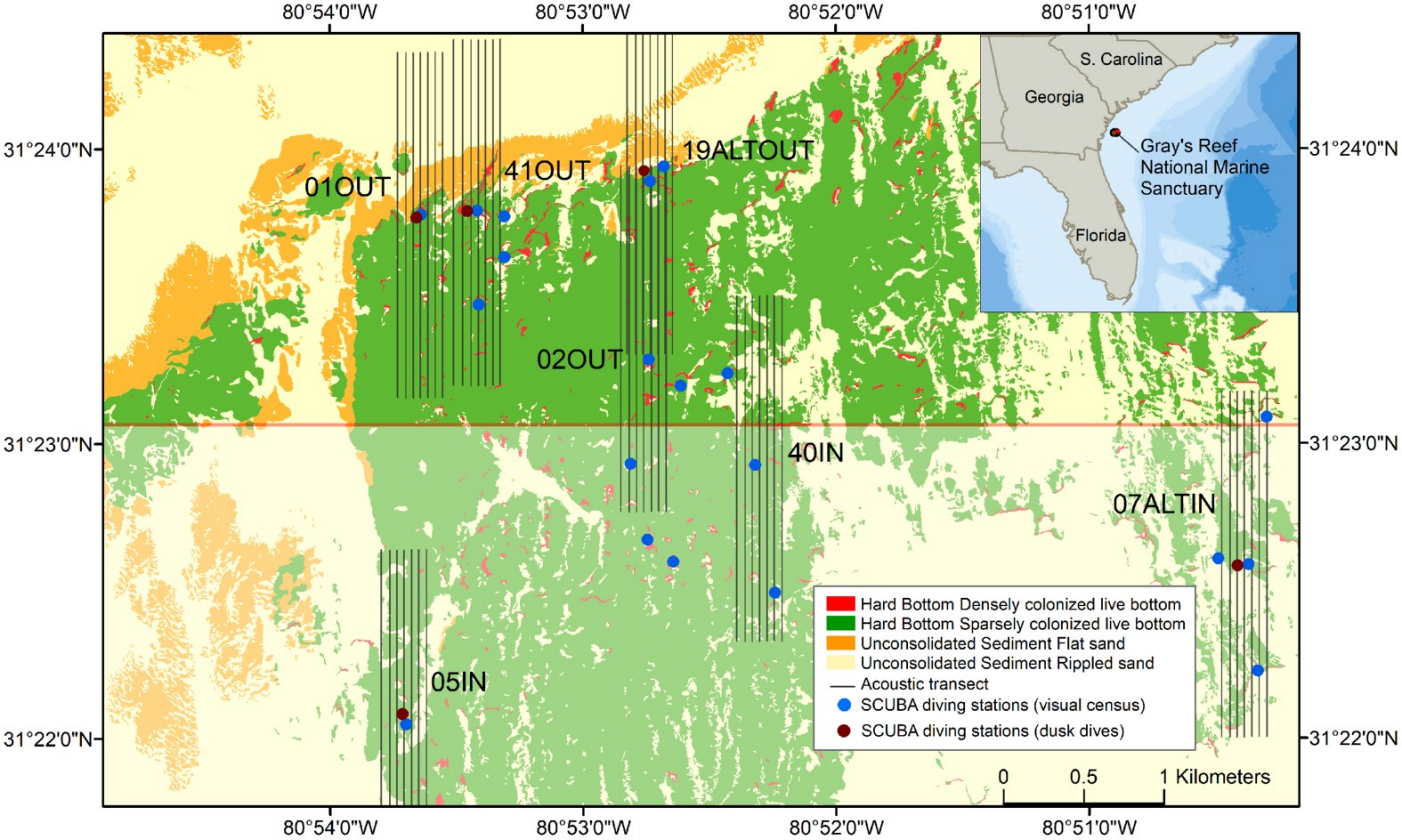
Habitat map of the study area with location of acoustic transects and sites with direct visual observations. The shaded area indicates the designated area closed to all fishing.

All the sites that were surveyed during this work were distributed within the boundaries of the Gray’s Reef National Marine Sanctuary (GRNMS). GRNMS is located 16 miles offshore of Sapelo Island, Georgia, in the NW Atlantic, in the transition zone between temperate and tropical waters. The seafloor setting is composed of widespread sand flats and submerged sandstone with rock outcroppings and ledges, known as “live-bottom reefs”. Depths within GRNMS ranges from 14 to 21 m. Mapping efforts [32] have described the habitat with high spatial accuracy, revealing that unconsolidated sediments dominate the seafloor of this region, covering 75% of the total area, and that colonized hard bottom occurs over the remaining 25%. Densely colonized undercut ledges account for a fraction of 1% of the total area (Fig 1). The variability across the hard bottom habitat within the sanctuary is high with features characterized by flat, smooth surfaces, exposed vertical scarps and ledges with undercuts, crevices, and slopes providing microhabitats for diverse fish and invertebrate species [32,33]. GRNMS is divided into two zones regulating fishing activities. One is a designated Research Area closed to all fishing while the second adjacent zone is open to recreational hook-and-line fishing with species, season, and size regulations set by regional fishery management agencies. No anchoring or spearfishing is allowed anywhere within GRNMS.

The activities were conducted under the GRNMS Superintendent’s general permit to conduct research and monitoring activities in the Sanctuary.

### Sampling and data analysis

Acoustic data were collected from the NOAA Ship Nancy Foster in July 2016 using an EK60 split beam echosounder operating at 3 frequencies (38, 120, 200 kHz). Pulse length was set to 128 μs for the 120 kHz and 200 kHz and 256 μs on the 38 kHz. The system was calibrated before the cruise following standard methods using a tungsten carbide sphere [34]. We surveyed a total of 7 stations and at each station an acoustic survey was repeated 6 times over 24 hours (pre-dawn, post-dawn, daytime, pre-dusk, post-dusk, nighttime; approximately 1.5 hours each survey). The survey design consisted of seven parallel transects 1 nm (1.852 km) long and spaced 50 m apart (Fig 1). The vessel speed was approximately 6.5 knots. Approximately 7 nmi of track lines were surveyed for each of the six sampling cycles (ca. 50 nmi total) at 7 stations. Predator and prey densities were estimated for each of the surveys and the diel trend of fish density was standardized by sampling site.

The spatial and temporal variability in the study area was high. Even though there is some spatial overlapping between sites, we can consider all of them as independent samples because they were surveyed at different times and the main target of the surveys (high relief ledges) were spatially separated.

Acoustics data analysis was performed using the Echoview ver. 8 software (Echoview Pty Ltd., http://www.echoview.com) following workflows described in [29] and [30]. First, noise from ship systems and unwanted backscatter from bubbles and other sources were removed from the acoustic echograms, which are a two-dimensional representation of targets in the water column. Two different approaches were used to estimate fish density. High density backscatter (schooling fish) was analyzed using echointegration [35]. The schooling fish density was derived by scaling the total acoustic backscatter with the target strength (TS) detected at the edge of the school, assuming that it is representative of the fish within the school. The low density backscatter (fish with individual swimming behavior) was analyzed using target tracking and echocounting [29,35].

In all cases, the estimated fish densities were divided into three size classes (small < 11 cm TL, medium 12–29 cm TL, large > 30 TL) using TS as a proxy for fish length. A generalized TS—length relationship [36] was used to estimate fish size. Trophic guild status based on size was initially assigned from prior visual survey data of species composition, size distribution, and the spatial relationship of predators and prey (e.g., species-specific school shapes and responses) over the seafloor landscape (unpublished data, [33,37,38]). These patterns were confirmed with direct visual surveys by divers during the current study period. Predator guild fishes were associated with the largest size class individual fish while prey was associated with the small and medium size class schooling fishes. In addition, 3.9 hrs of observations focused on predator-prey behavioral interactions were made during evening crepuscular periods (10.2 hours overall across daylight through twilight periods) that facilitated interpretation of acoustic records and identified those interactions that served as drivers of changing fish distributions. Additional daytime visual fish census dives (n = 20) were performed at stations located in the proximity of the acoustics survey sites that were used to describe species composition of functional groups (pelagic predators, demersal predators, prey). Mobile conspicuous fishes were assessed by conducting visual census with standardized 50 x 10 m transects, while cryptic and juvenile prey species <10 cm TL were targeted with 25 x 2 m transects (sensu [39]).

This observational study was exempt from requirements for an approved protocol from an animal ethics committee. No samples were collected and observation methods did not interfere with behaviors or manipulate the environment.

### Habitat suitability modeling

Maximum entropy species distribution models (MaxEnt) were used to investigate diel variability in patterns of habitat selection by prey and predator fish. MaxEnt is implemented using presence-only occurrence records and pseudo-absences data as background information [40]. The species probability distribution is estimated by finding the probability distribution of maximum entropy, which is closest to uniform and constrains the distribution to a set of environmental variables that describe habitat characteristics based on the distribution of the species. Variable selection and overfitting are controlled by regularization (L1- regularization, [41]).

Response variables were the occurrences of predators and prey fish estimated from the analysis of the acoustic survey data. Predictor variables were habitat metrics derived from a recent multibeam survey carried out in the GRNMS that describe seafloor topography and complexity (rugosity, slope, slope of the slope bathymetry, curvature; see Table 1 for a complete list of predictors and detailed descriptions). The habitat metrics were used with their original resolution (2 m). Moreover, considering that predator and prey species can be influenced by the habitat over larger ranges, we also derived lower-resolution predictors computing the 3rd quartile within a radius of 50 m for each of the variables.

**Table 1:** Descriptions of the habitat metrics used as predictors in the MaxEnt models.

Predictor	Description	Unit
Depth	Water depth	Meters
Depth (Standard Deviation)	Dispersion of water depth about the mean (3x3 cell neighborhood)	Meters
Slope	Maximum rate of change in slope between cell in a 3x3 neighborhood	Degrees
Slope of the slope	Maximum rate of maximum slope change between cell (3x3 cell neighborhood)	Degrees of degrees
Curvature	Rate of changes in curvature across the surface (3x3 cell neighborhood)	Concave (-), Convex (+)
Planar Curvature	Curvature of the surface perpendicular to the slope direction (3x3 cell neighborhood)	Concave (-), Convex (+)
Profile Curvature	Curvature of the surface in the steepest down-slope direction (3x3 cell neighborhood)	Concave (-), Convex (+)
Rugosity	Ratio of surface area to planar area (3x3 cell neighborhood)	Ratio

Model validation was performed on a validation dataset, which was created by taking a random selection from the original dataset (25% of the total data). Receiving operating curves (ROC) were estimated and Area Under the Curve (AUC) was used to assess the model performances [42].

Habitat suitability maps were constructed based on the prediction of the models for both predators and prey over the entire area of the sanctuary. The predicted continuous probability of suitability was converted into two classes (suitable and unsuitable habitat) based on a threshold probability. The threshold was estimated for each model using the MaxSSS method which maximizes the sum of sensitivity (proportion of presences correctly predicted) and specificity (proportion of absences correctly predicted). This threshold was previously demonstrated to produce the most accurate predictions [43,44]. MaxEnt was performed using the standalone java application (version 3.4.1; http://biodiversityinformatics.amnh.org/open_source/maxent).

### Spatial analysis

The spatial patterns of distribution of predators and prey were investigated using a set of spatial indicators calculated using the acoustic-estimated fish density. Specifically, we estimated center of gravity, positive area, spreading area, equivalent area, inertia and a global collocation index (Table 2) for both predators and prey and compared their temporal variability. Positive area measures the space occupation of the fish densities that are greater than zero without taking into account differences between low and high densities. Spreading area, similar to the positive area, measures the area occupied by the population but taking into account the variation of fish densities. Equivalent area is an individual-based model of spatial coverage of the population and assumes that all individuals have the same density, which is equal to the mean density per individual. Inertia is a measure of the dispersion of the population around its center of gravity and it can describe how the population is scattered across the spatial domain. The global collocation index describes the spatial overlap between two different populations measuring the proximity of the two centers of gravity, given the dispersion of each population (inertia). The index ranges from 0 when each population is concentrated in a single but different location to 1, when the two centers of gravity fully overlap. A full description of the indicators can be found in [45]. In order to take into account possible differences between sampling sites, the spatial indicators were standardized by site subtracting the mean and dividing by the standard deviation. Analysis was performed using R v. 3.4.1 (R, 2012). The spatial bin used to calculate the indicators was 50 m. In addition, to investigate potential diel changes of prey schooling behavior, the temporal variability of prey fish school size (area) and packing density were also computed and compared. Modification in schooling behavior can indicate a response to external stimuli such as changes in environmental conditions (e.g., light, turbidity, zooplankton prey) or presence of predators.

**Table 2:** Spatial indicators used in the study.

Spatial Indicator	Description	Formula	Unit
Positive area (PA)	Area occupied by the population when the density is > 0	$\sum_{i}s_{i}\;1_{z_{i}>0}$	Km^2^
Spreading area (SA)	Area occupied by the population accounting for variation in density	$2\int \frac{Q-Q(T)}{Q}dT$	Km^2^
Equivalent area (EA)	Individual-based index of the area occupied by the population	$\frac{Q}{\int z(x)(\frac{z(x)}{Q})dx}$	Km^2^
Center of Gravity (CG)	Mean location of the population	$\frac{\int_{i=1}^{N}x_{i}s_{i}z_{i}}{\int_{i=1}^{N}s_{i}z_{i}}$	location (lon, lat)
Inertia (I)	Dispersion of the population around its Center of Gravity	$\frac{\sum_{i=1}^{N}{\left(xi-CG\right)}^{2}s_{i}z_{i}}{\sum_{i=1}^{N}s_{i}z_{i}}$	adimensional (0–1)
Global Index of Collocation (GIC)	Measure of the overlapping between two populations	$\frac{\Delta CG^{2}}{\Delta CG^{2}+I_{1}+I_{2}}$	adimensional (0–1)

**z(x)**: density of population at location x; **s**_**i**_: area of influence (area made up of points in space that are closer to this sample than the others; **Q**: overall abundance; **T**: cumulated area occupied by densities values

In order to evaluate the potential effects that daily changes in light levels can have on the spatial distribution and behavior of predators and prey, Photosynthetically Active Radiation (PAR, mmoles/m^2) collected over the entire time of the survey was analyzed. The PAR data were obtained from a weather station at Marsh Landing on Sapelo Island-Georgia, located 16 miles west of the study area [46].

## Results

**Fig 2:**
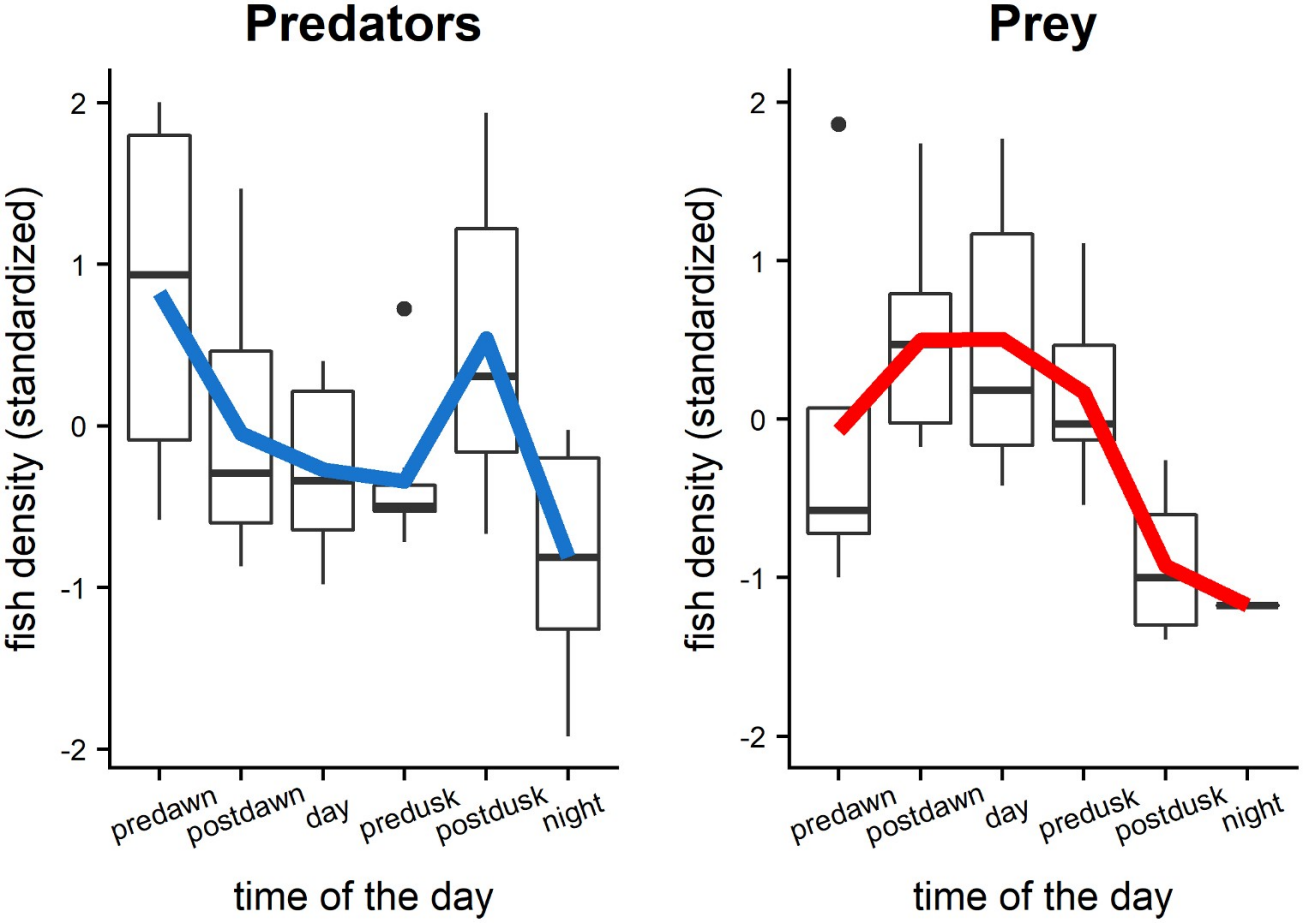
Boxplots of prey and predator fish densities describing diel trends from the acoustic surveys by time period. Colored lines represent the average fish density across sites. Lower and the upper hinges correspond to the first and third quartiles. Dots at the end of the boxplot represent outliers.

The site-standardised density of predators and prey exhibited strong opposing diel trends except at night when both predators and prey were at extremely low densities (or not detected) (Fig 2). In particular, predator density was highest during the crepuscular period (dawn and dusk), while prey fishes had a peak in density during the day and reduced levels at dawn and dusk. Even though densities were standardized by sampling site to minimize the effect of differences in site characteristics (e.g. habitat, species composition), a degree of variability within sample periods was also observed.

The diel variability of prey density detected was consistent with the general diel characteristics of fish behavior we observed in the echograms (S1 Fig). Small prey fish dispersed during the night in the water column, then at dawn were observed to aggregate, forming dense and organized schools, and moved toward hard bottom habitats. The prey fish schools remained in dense aggregations throughout the day and then transitioned to looser aggregations at sunset moving up and dispersing again away from ledges (S1 Fig).

Cryptic species, those not readily detected by the echosounder, were not included in this study. The most abundant predator species observed, based on relative abundance, was greater amberjack—*Seriola dumerili* (pelagic, 56%) and black sea bass—*Centropristis striata* (demersal, 88%). Prey species were mainly composed of tomtate—*Haemulon aurolineatum* and scad—*Decapters* sp. representing 97% of the total prey fish observed (Table 3).

**Table 3:** Species composition divided by functional groups estimated from the daytime visual census surveys.

Pelagic predators			Demersal predators			Prey		
Species	Common name	%	Species	Common name	%	Species	Common name	%
*Seriola dumerili*	Greater Amberjack	56	*Centropristis striata*	Black Sea Bass	88	*Haemulon aurolineatum*	Tomtate	83
*Sphyraena barracuda*	Great Barracuda	19	*Lutjanus campechanus*	Red Snapper	9	*Decapters sp*.	Scad	14
*Seriola rivoliana*	Almaco Jack	12	*Mycteroperca microlepis*	Gag Grouper	2	*Rhomboplites aurorubens*	Vermillion snapper	3
*Scomberomorus maculatus*	Spanish Mackerel	12	*Lutjanus synagris*	Lane snapper	0.7			
*Carangoides bartholomaei*	Yellow Jack	1	*Mycteroperca phenax*	Scamp Grouper	0.3			

The % column corresponds to the overall percentage of the species for the whole study area.

**Fig 3:**
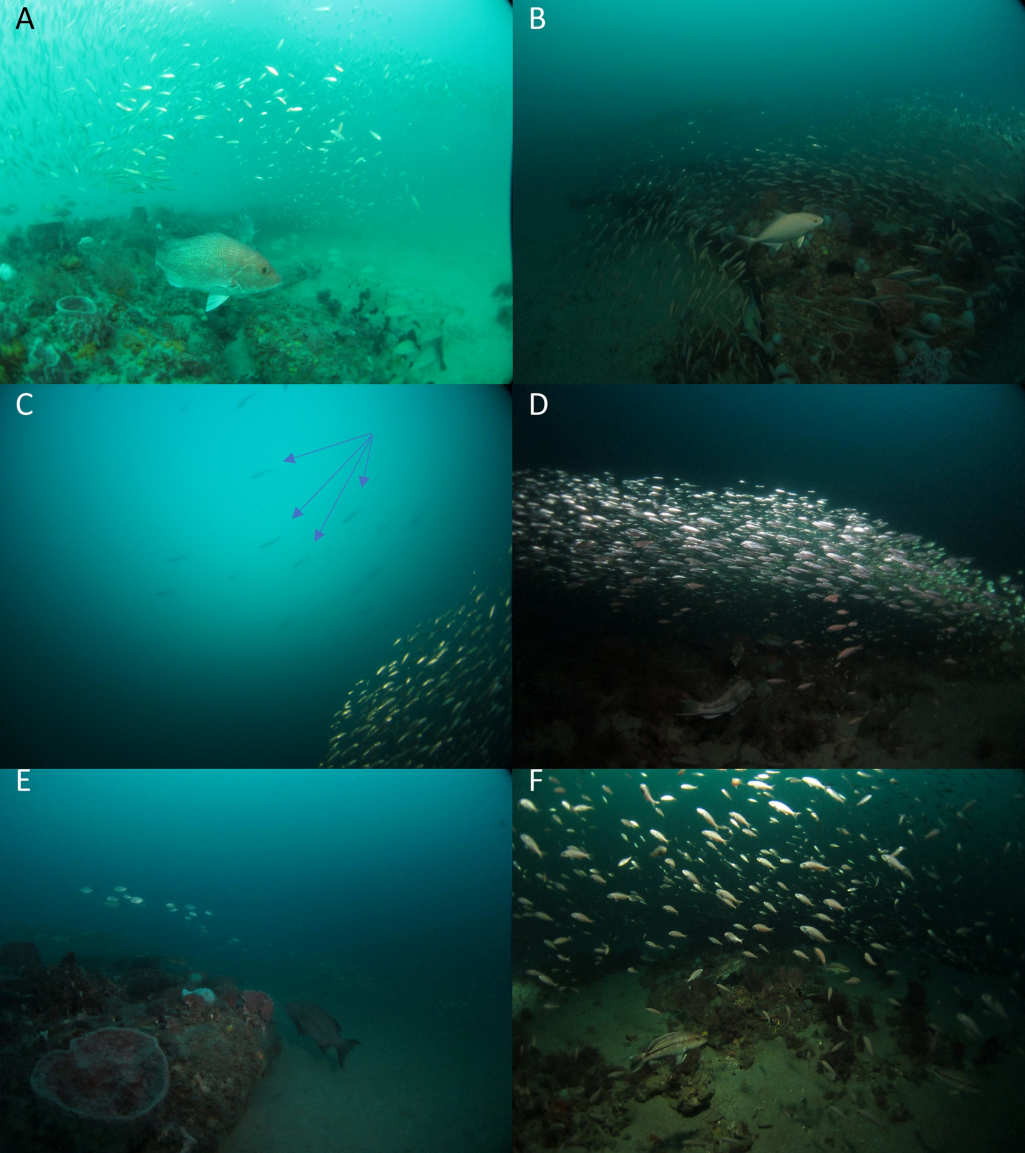
These images depict predator-prey interactions via active stalking and attack. (A) Example of daytime reef with prey school of Decapturus sp. compressed towards reef by attacks from above by mid-water predators and stalked under school by red snapper, leading to subsequent attack. Almaco jack (B) and Spanish mackerel (highlighted by the blue arrows) (C) attacking prey school from above at dusk. (D) Black sea bass at dusk attacking compressed school of Decapturus sp. in tubular formation fleeing from mid-water predators. (E) Scamp grouper at dusk stalking compressed school of prey. (F) Black sea bass attacking prey (juvenile Tomtate) rising above reef before dispersal after twilight.

Diving observations during the evening crepuscular period facilitated a descriptive view of the continuous predation activity and prey response at small space and time scales (Fig 3). There was a general pattern of reduced horizontal extent of dense prey fish aggregations with fish concentrating along the deep undercut edge of high relief reefs about 20 minutes prior to sunset through twilight, with fish still oriented to the undercut edge and adjacent live-bottom habitat, but rising above the seafloor (with seafloor-associated aggregations ca. 1m and ranging 5+ m vertically into the water column) before rapid dispersal post-twilight. In the absence of mid-water predators attacking prey aggregations from above and sides, prey species rapidly dispersed at the approximate time the upper disk of the sun disappeared below the horizon (time of prey dispersion observed by divers and ca. time of apparent sunset determined by NOAA Earth System Research Lab Sunrise/Sunset Calculator for a geographic position central to GRNMS https://www.esrl.noaa.gov/gmd/grad/solcalc.html; accessed on 5 July 2016).

During the period of reduced ambient light at the seafloor (ca. 20 minutes before sunset), demersal piscivores attacked aggregations and schools of prey from below and along the sides of aggregations, causing them to rise. Attacks by mid-water predators from above appeared to be the proximate driver of continued presence of prey aggregations on reefs after sunset, with dispersal coincident with cessation of predation. The escape responses of prey from the mid-water predators facilitated the continued predation by demersal piscivores after sunset.

### Habitat suitability modeling

MaxEnt models were parameterized for both predators and prey for each time period, except for the night surveys where the number of occurrences was not large enough to produce reliable model results (Table 4). All models had an AUC greater than 0.6 indicating that they are predicting better than a random model. However, the AUC cannot be used in presence-only models to compare the accuracy of different models because the maximum value of AUC is not known, it varies based on the true distribution range of the modelled species, and consequently we cannot determine how close to optimal a given AUC value is [40]. For this reason, the lower AUC values of the predator models do not necessarily mean that the performance of the models are poor but, instead, could be related to the wider habitat preference of those species.

**Table 4:** Characteristics and variable importance of the Maxent models for predators and prey.

MAXENT models		Predators					Prey				
		Predawn	Postdawn	Day	Predusk	Postdusk	Predawn	Postdawn	Day	Predusk	Postdusk
**Model characteristics**	n occurences	209	137	116	115	131	51	139	161	139	107
	AUC (training)	0.668	0.635	0.722	0.676	0.622	0.854	0.778	0.792	0.812	0.744
	AUC (testing)	0.614	0.659	0.664	0.702	0.608	0.840	0.805	0.720	0.828	0.635
	maxSSS	0.58	0.63	0.39	0.51	0.59	0.41	0.49	0.29	0.36	0.59
**Variable importance**	Depth SD	0.00	0.00	0.00	0.00	0.00	0.00	0.87	0.93	2.77	0.60
	Depth SD (q)	**12.41**	8.45	1.55	0.00	8.14	0.02	3.63	**20.84**	2.04	2.53
	Slope	0.00	0.00	0.14	0.65	0.00	0.09	2.11	0.08	0.18	0.48
	Slope (q)	0.01	**10.52**	0.74	5.59	2.00	0.13	**25.61**	2.69	5.39	1.27
	Slope of slope	0.02	3.71	8.01	4.27	3.49	0.94	0.60	1.42	1.20	0.77
	Slope of slope (q)	**20.57**	**32.55**	**70.14**	**56.18**	**45.48**	**56.97**	**48.21**	**65.91**	**75.94**	**51.54**
	Curvature	0.00	0.11	0.00	0.00	0.00	0.00	0.00	0.00	0.13	0.49
	Curvature (q)	**19.38**	6.43	1.02	0.00	6.81	2.03	4.55	1.56	3.29	9.85
	Planar Curvature	0.00	0.72	0.00	0.00	0.00	0.00	0.01	0.02	0.59	0.00
	Planar Curvature (q)	0.00	4.47	0.52	0.68	4.94	**23.71**	2.11	2.46	1.15	**15.19**
	Profile Curvature	0.00	0.00	0.45	1.25	0.00	0.02	0.00	0.00	0.00	0.04
	Profile Curvature (q)	0.18	4.36	2.54	0.25	0.75	**11.15**	2.52	1.31	0.49	4.09
	Rugosity	2.01	7.68	0.52	5.95	0.23	0.00	4.66	0.01	1.35	0.00
	Rugosity (q)	**17.52**	**11.69**	4.69	**11.11**	6.22	4.94	5.12	2.78	5.47	**13.16**

Bold values correspond to the parameters with a relative importance for the model greater than 10%. The variable name followed by q indicates the 3^rd^ quartile of the original variable calculated in a 50 m radius

**Fig 4:**
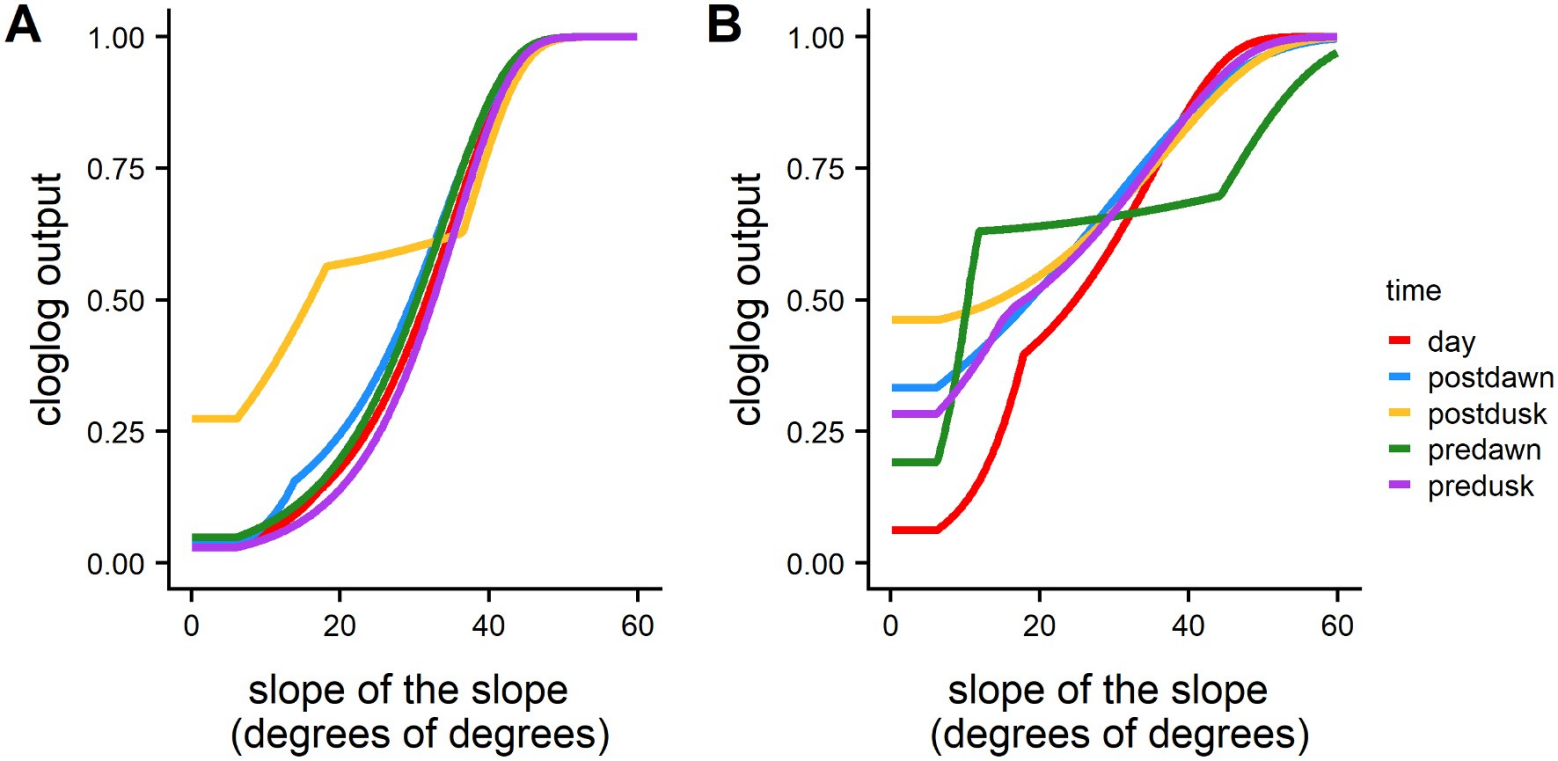
Partial response curves of slope of the slope variable for all the prey (A) and predator (B) models. The complementary log-log (cloglog) value on the y axis can be interpreted as the predicted probability of presence.

For both predators and prey, the habitat variables with the lower resolution (3rd quartile of the variable within 50 m radius) contributed the most in the model estimation. In particular, slope of the slope was the most significant variable for all the models followed by rugosity (Fig 4). Both predators and prey generally showed a clear preference for high values of slope-of-the-slope that correspond with areas of higher local-scale relief. Predator response curves presented a less steep slope compared to the prey curve, indicating a preference for a wider range of values.

**Fig 5:**
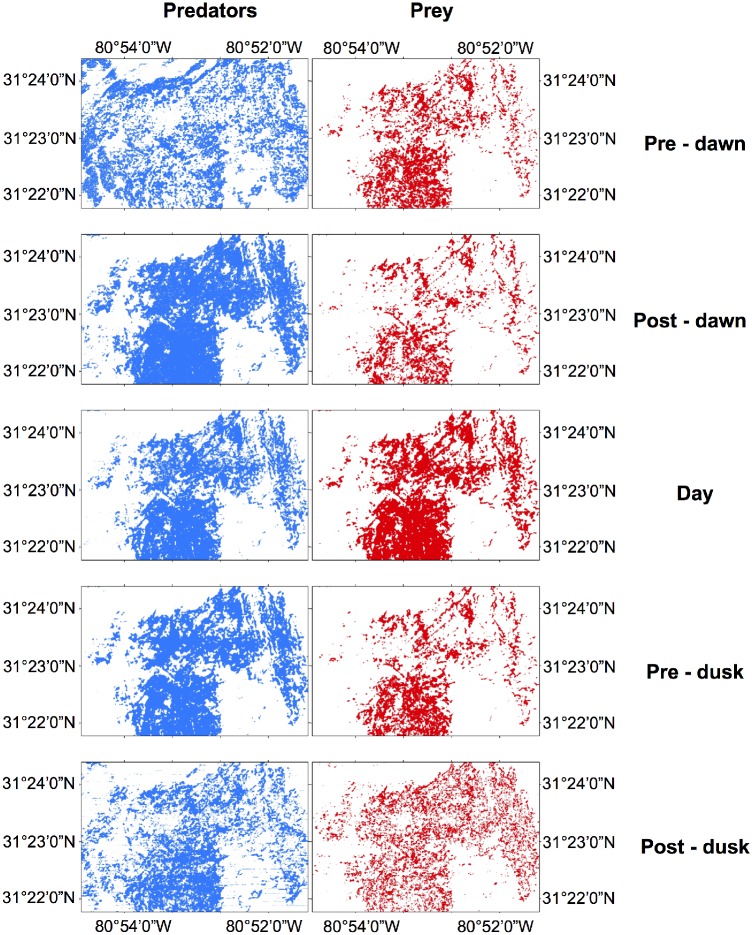
Prediction maps of high and low habitat suitability for predators and prey estimated from the MAXENT models. The maps were obtained thresholding the continuous prediction of probability of suitability using the maxSSS (maximum sum of sensitivity and specificity). Blue and red represent high habitat suitability and white low habitat suitability.

**Fig 6:**
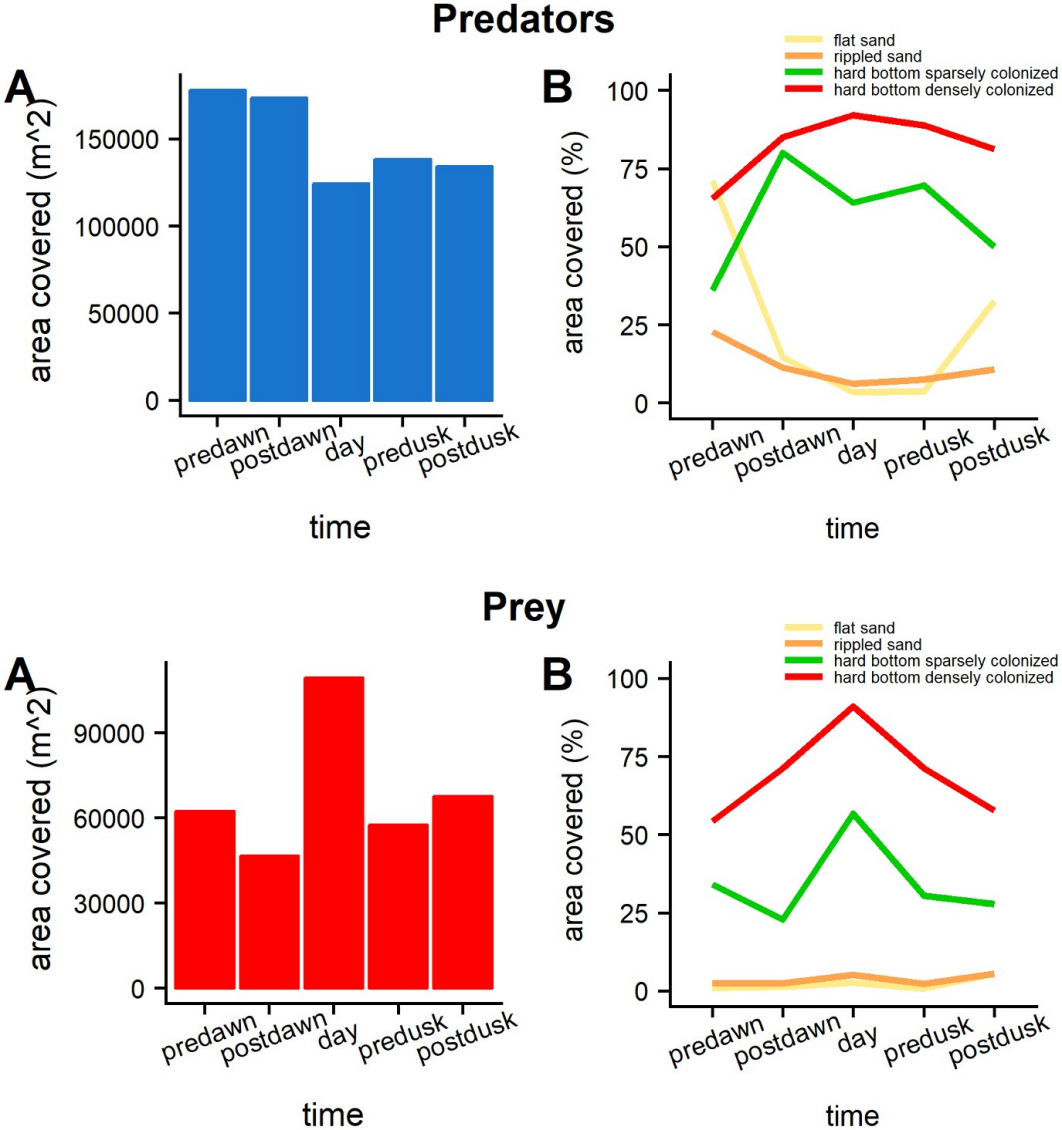
Extent and habitat type of high habitat suitability predictions. A) Total area covered by the high suitability predictions by time interval. B) Percentage of habitat separated by habitat type, covered by the high habitat suitability predictions.

Maps of habitat suitability, estimated for the whole sanctuary, illustrate the spatial variation in the extent of highly suitable habitats for both predators and prey over the diel period (Fig 5). The differences between twilight periods and daytime are especially pronounced. The area covered by the suitable habitat of predators was greater during dawn and dusk, with a greater area of occupation than the daytime (Fig 6). The suitable area over sandy habitats increased considerably at predawn and postdusk along with a decrease of suitable areas over hard bottom. The area covered by suitable habitat for prey is smaller than that for predators throughout the whole diel cycle and is larger during the daytime compared to the rest of the day. The suitable habitat for prey occupied almost exclusively hard bottom habitat especially during the daytime where over 90% of the habitat classified as “hard bottom densely colonized” was predicted as highly suitable.

**Fig 7:**
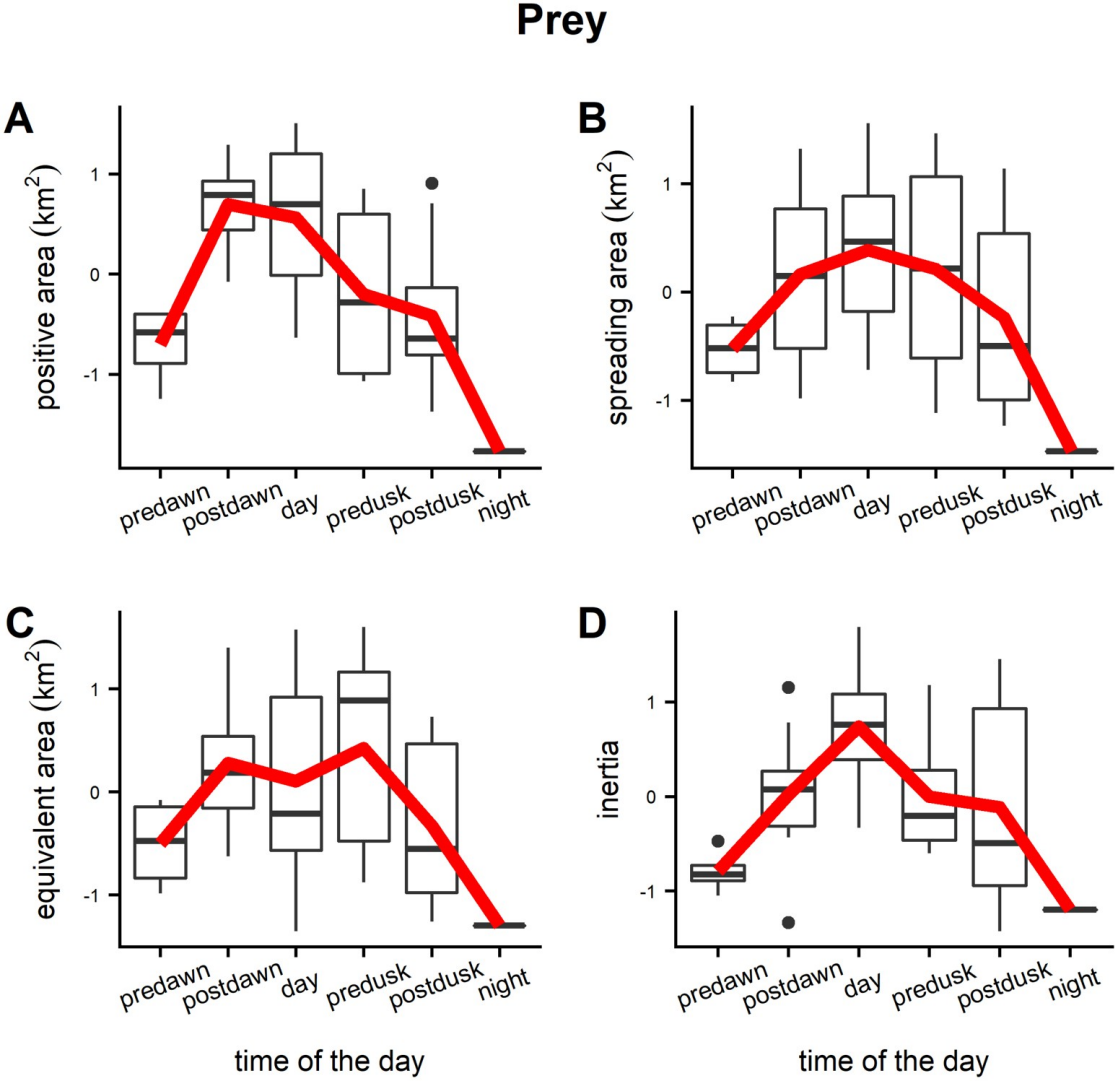
Boxplots of standardized spatial indicators (positive area, spreading area, equivalent area, inertia) estimated using prey fish acoustic density. Red lines correspond to the average value of the indicators across the survey sites.

**Fig 8:**
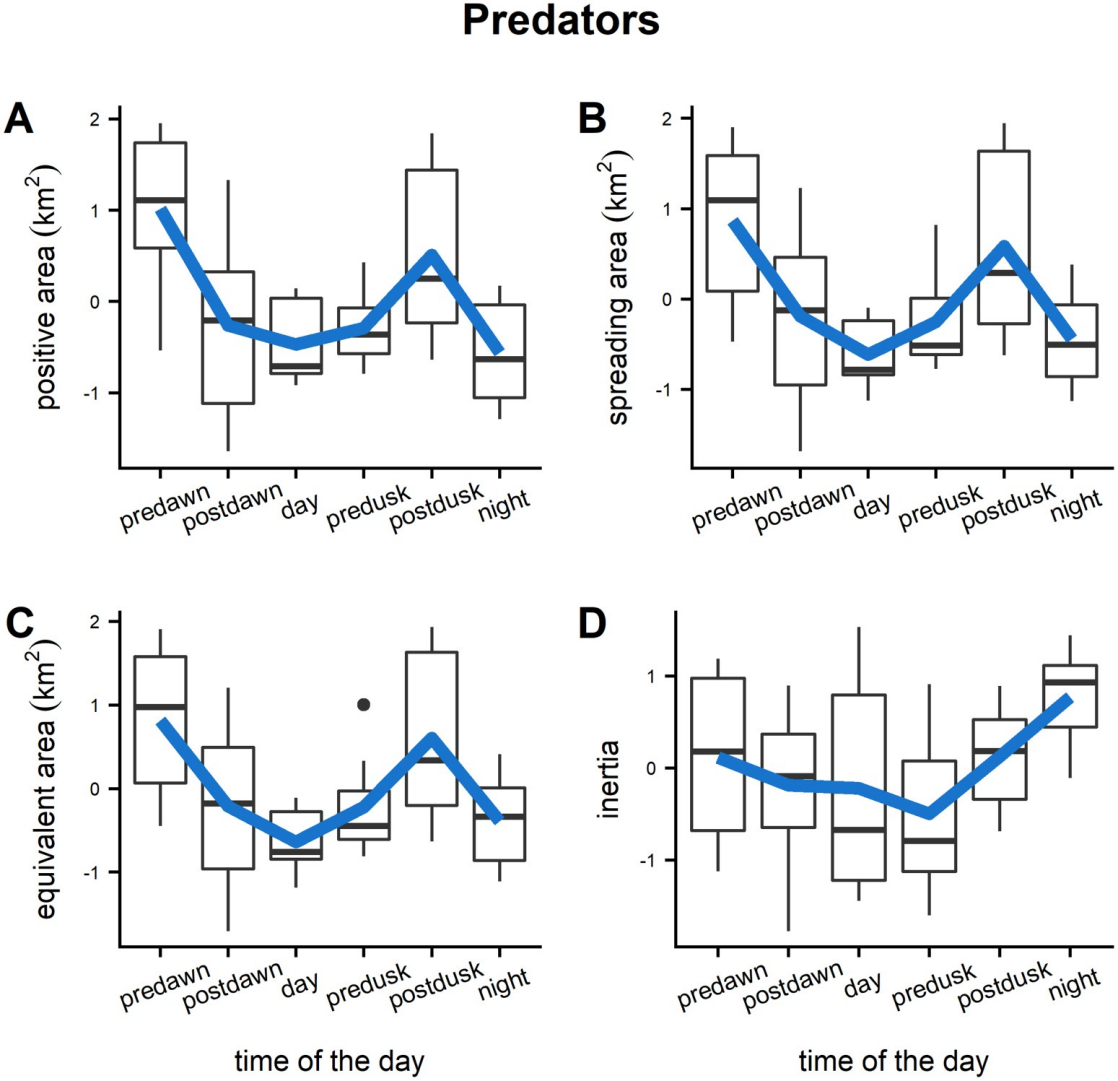
Boxplots of standardized spatial indicators (positive area, spreading area, equivalent area, inertia) estimated using predator fish acoustic density. Blue lines correspond to the average value of the indicators across the survey sites.

**Fig 9:**
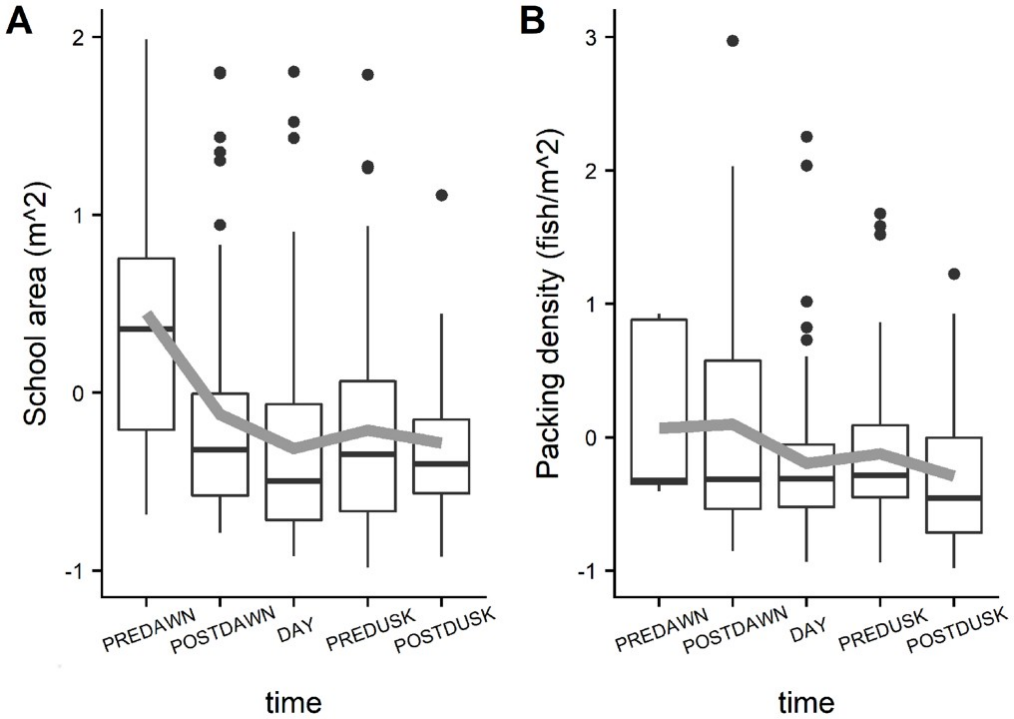
Boxplots showing diel trend of prey school area and packing density from the acoustics survey by time period.

**Fig 10:**
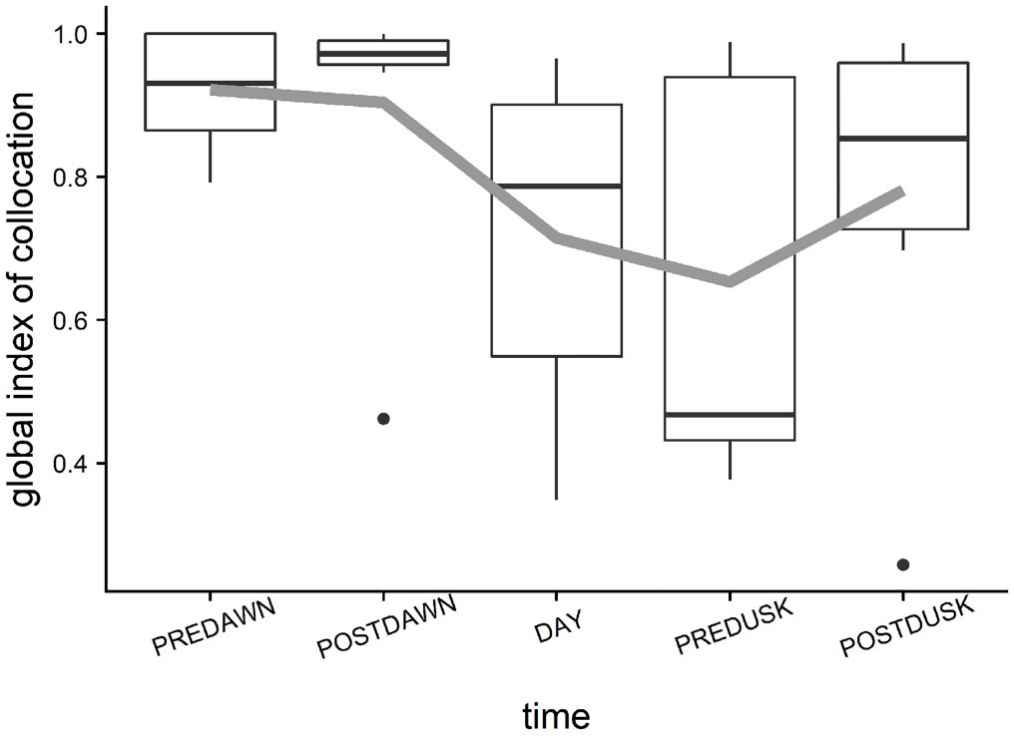
Boxplots showing diel trend of global collocation index. It ranges between 0, where the 2 populations are concentrated on a single but different location, and 1, when the two centers of gravity coincide. Gray line corresponds to the average value of the indicator across the survey sites.

Diel trends of averaged positive area, spreading area, equivalent area and inertia for the prey presented a similar pattern with the highest values reached during the daylight hours (Fig 7). This indicates that during daytime prey had a larger spatial range and were more dispersed around their center of gravity. The fact that positive area and spreading area followed a similar pattern denoted that high fish density values contributed to the increase of the spatial range of distribution. During the crepuscular periods the spatial indicator values decreased indicating that the spatial extent of prey distribution was lower and with a low dispersion around the center of gravity. The values of spatial indicators calculated at night for prey could not be realistic considering the few number of schools observed during that period. Predators showed an opposite trend (Fig 8) with a larger dispersion and spatial distribution extent during the twilight periods. Diel variation of prey school size and school packing density were also evaluated (Fig 9). Large variation was detected for both of the parameters especially for the packing density where no clear trends were detected. Prey school area was larger on average at pre-dawn, but no other large differences were detected during the rest of the day. The larger values of the Global Collocation Index were found at predawn and postdawn (average GIC 0.92, 0.90 respectively) indicating high degree of overlapping between predators and prey (Fig 10). The lowest level of overlapping occurred during the daytime (average GIC: 0.71).

**Fig 11:**
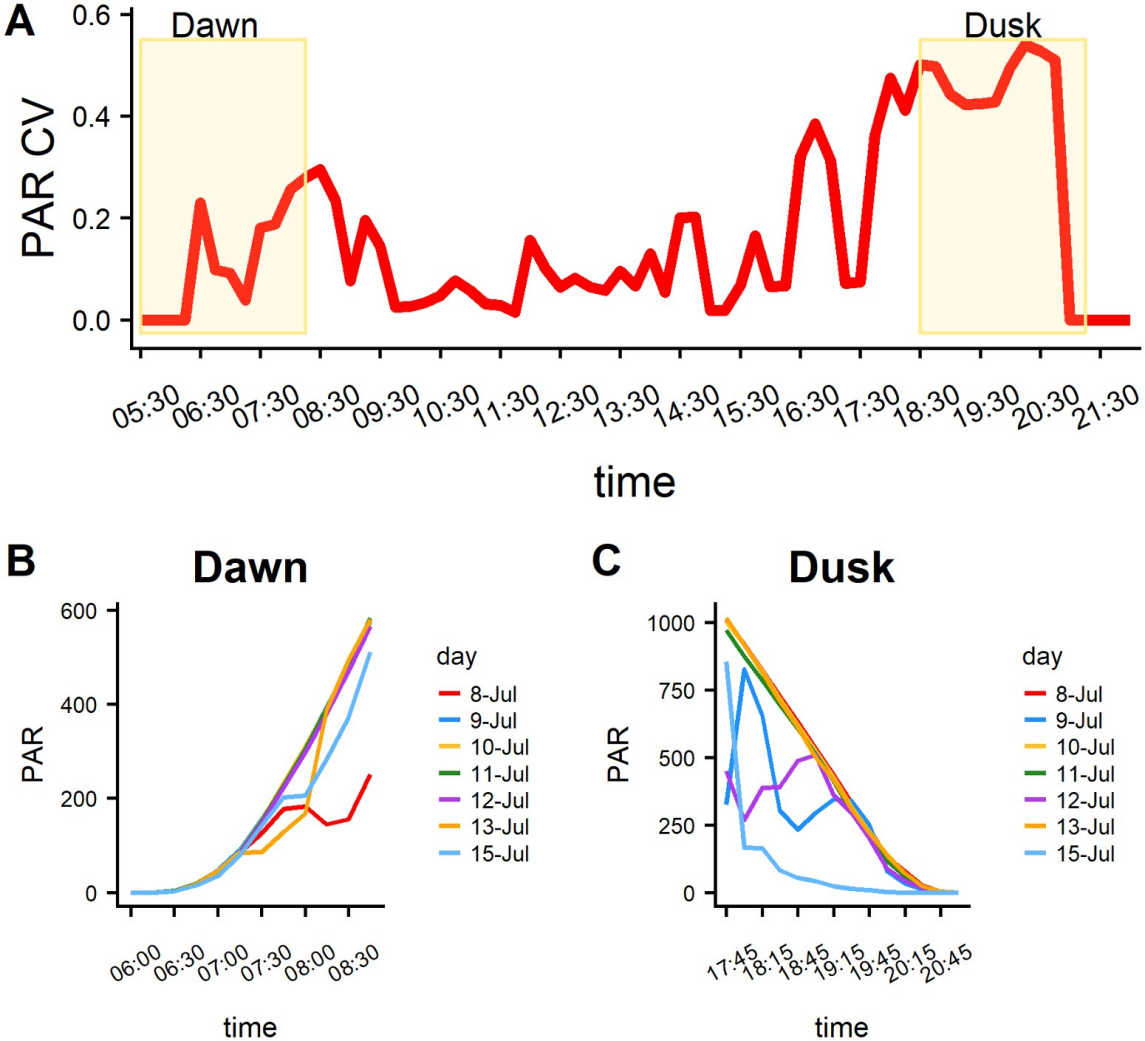
Photosynthetically Active Radiaton (PAR) data. A) Diel temporal trend of CV (Coefficient of Variation) of PAR. The shaded areas corresponded to the crepuscular phases. B, C) Day-to-day trend of PAR during the crepuscular phases.

The temporal trend of PAR (Photosynthetically Active Radiation) over the survey period, and especially over crepuscular periods, is shown in Fig 11. PAR presented a higher variability during the crepuscular period, in particular at dusk. This general pattern can be linked to the higher variability in fish behavior at dusk in terms of both prey dispersal and predation intensity (i.e., based on number of predators and group behavior quantified in acoustic surveys).

## Discussion

Our results demonstrate that prey and predator species exhibited complex spatial dynamics and behavior over 24 hour periods, with prey modifying patterns of habitat use and spatial distribution, likely as a response of their interactions with predators. Acoustics, direct visual observations, and model results provided complementary sources of information that support the inferences of predation risk-driven habitat selection behaviors of prey in this sub-tropical reef setting (sensu [47]).

### Predator and prey behaviour

The behavioral patterns observed by the acoustic surveys are characterized by an alternation between a more aggregated phase (fish organized in schools) during the daytime where most of the zooplanktivorous prey species are more active and detectable, and a dispersed phase during the nighttime where few fish were detected. These periods are linked by two crepuscular phases (dawn and dusk) where an increase of direct interactions between predators and prey occur. This overall pattern is well documented in diverse ecosystems and fish communities [25] with multiple studies highlighting the importance of crepuscular periods as a behavioral, ecological and environmental transition [25,48,49,50].

In this study, we observed that predator fish density increased during the crepuscular period (around sunrise and sunset), coincident with a peak of predation activity. During the twilight period predators can take advantage of the vulnerability of prey that are transitioning in regard to density and patch size. For example, through dusk prey fish transitioned from dense and organized daytime schools through a period of loose aggregation and ultimately wide dispersal (and vice versa during dawn). Indeed, some predator species have greater visual acuity under low and intermediate levels of light that is related to the composition of retinal pigments, adding a visual advantage over the prey [51,52]. Predator density (at least for non-cryptic predators) was comparatively low during the daylight period, although predation on prey fish does occur [38]. On the other hand, schooling prey fish presented an opposite trend, with the lowest density (before the nocturnal dispersal from the reefs) during dawn and dusk and the highest during daytime. This pattern may be a response to the diel variation of predation activity. Prey may have a lower perception of predation risk, at least per capita, during the day and forage in areas where accelerated flows over reefs deliver prey at an increased rate compared to surrounding areas with lower local relief [53]. Spatial indicators and the results of the habitat modeling also confirmed this hypothesis.

### Spatial analysis and habitat suitability modeling

The large values of all the area indicators and the inertia for the prey during the daylight hours, suggest that there is an increase of their distributional range. Moreover, the habitat modeling results illustrate that the habitat suitability for the prey increased considerably during the day with an increasing preference for lower-relief habitats. The high activity level of predators was also a clear pattern revealed by inspection of the suitability maps and the spatial indicators. The spatial extent of predators increased during the crepuscular periods. Predators occupied a larger range of habitats including also sandy habitats indicating that they might be actively searching for prey. Another indication of a predation peak during crepuscular periods is higher values of GIC (Global Index of Collocation) indicating a higher overlap between predators and prey distributional range during that time.

These results are in agreement with the theory of the “landscape of fear” [16,54]. According to this theory prey behavior is shaped based on the distribution of predation risk across the habitat mosaic. Different types of habitat have different levels of risk based on presence of refugia, escape probability and likelihood of predator attack. Prey then select from these different habitats based on their perception of risk. The relative size of the domain, habitat complexity, and patchiness of GRNMS appear to allow prey to move easily from different habitats in short periods of time, altering their habitat use according to the characteristics of the seafloor and avoiding areas assumed to be high risk of predation.

All of the MAXENT model results illustrated that preferred habitat for both predators and prey is characterized by a high level of topographic complexity (high relief ledges). Slope of slope was the best explanatory parameter in the computation of the models indicating this metric represents well the differences between habitats across the study area. Slope of slope was also found to be an important measure of seafloor habitat variability in other studies that modeled fish distribution in coral reef ecosystems [29,55]. The higher importance of the parameters that integrate across a broader spatial scale (i.e., 3d quartile predictors) can be related to the dynamic nature of associations of species with high movement rates to specific habitat patches (e.g. predators: barracuda, amberjack; prey: scad). It is important to point out that the MAXENT models estimated habitat preferences for two groups (predators and prey) that include multiple species and therefore some of the species could have diel movement characteristics that differ from the general outcomes of the models.

### Drivers of variability

Even though the overall patterns inferred by the analysis of the acoustic data are clear, the degree of variability was substantial, especially at dawn and dusk. In particular, surveys conducted at dusk presented the highest level of variability across sites that are also detectable in the outcomes of the spatial analysis. In order to eliminate the variability generated by the different level of fish densities across sites, we used standardized values to explore the temporal trends of fish density and spatial patterns. Hence, the variability detected is related to other behavioral and external factors. Many studies have highlighted that fish exhibit a degree of plasticity in their diel rhythm that can be related to developmental (e.g. ontogeny, spawning behavior) and environmental aspects (e.g. temperature, light, food availability) [56]. In this study we identified several local factors that could have acted synergistically to generate this variability and are related to the transitional nature of the crepuscular phases. Light is one of the natural elements that drive these transitions. Variation of ambient light levels due to cloud cover, turbidity or moon phases could modify the behavior of predators and prey affecting, for instance schooling behavior and fish movement [56]. During the period of the study, the day-to-day variability of light levels was high during the crepuscular period, especially at dusk. This temporal trend corresponded to the patterns of variability observed in the spatial indices, indicating a potential large scale effect of light variation on the distributional patterns and interactions of predators and prey. More specific studies should be carried out to confirm these findings and explore specific localized effects induced by daily variation in the light regime that we were not able to identify within this study.

Other factors that could have increased the variability in the results might be related to fine temporal and spatial scale events that were not detected by acoustic methods alone. The use of direct observations via diving helped us to overcome this limitation and highlighted important behaviors. In particular, facilitative interactions between transient and resident piscivores were observed during the period of reduced ambient light prior to and during the dusk twilight period. This synergistic predation has been observed before in the same study area [38,57] and in other similar ecosystems [58,59]. The presence of such behavior could have a potentially large effect on population dynamics, for instance by increasing prey vulnerability and mortality. Hixon and Carr [58] observed that predators in a coral reef community induced clear density-dependent prey mortality only when demersal and pelagic predators co-occurred due to the lack of refugia for the prey that have to escape from two simultaneous predator attacks. At GRNMS, predation activity continued well after dusk when the presence of both classes of predators were present, having a direct effect on prey schooling behavior. Prey, in fact, delayed their dispersal at night as a defensive behavior until the transient predators moved away from the ledges. In the absence of mid-water predators attacking prey aggregations, prey species rapidly dispersed at the approximate time the upper disk of the sun disappeared below the horizon. This indicates that predation risk can largely vary across the area depending on the predator species composition. Prey then have to modify their distribution and behavior in order to respond to the predation threat. In our work, we did not analyze the direct effect of synergistic predation on prey mortality, and more work is certainly needed to better quantify the local and regional effects of this process on the demography of these species.

Since the use of an escape strategy or the use of refugia is limited due to the multiple directions of attacks from predators during the twilight period, remaining organized in schools appears to be the most effective defensive strategy. There are several mechanisms that have been proposed to explain the anti-predator advantage of being in a school rather than swimming independently. For instance, predators could experience a “confusion effect”, which is the difficulty of discerning and targeting a single individual within a school [60]. The presence of “many eyes” in a school could also increase the efficiency in detecting a predator that allow individual prey to reduce the general level of vigilance and dedicate more time to feeding [61,62]. Larger size schools potentially benefit more of these advantages over smaller groups. Prey school size measurements obtained from the acoustic survey presented larger values at dawn, and to a lesser extent at dusk, compared to the average size measured during the daytime, indicating prey can form larger schools during the peaks of predation activity.

## Conclusions

The results of this study highlight the effectiveness of the use of fisheries acoustics in association with direct observation to detect patterns of variation of predators and prey distribution and their interactions in a sub-tropical reef setting. The majority of the studies carried out in these areas relied on visual observations with limited spatial and temporal resolution or on ex-situ experiments [2] but they failed in many cases to scale-up the results to larger areas. Acoustics has the advantage of providing high resolution data (both spatial and temporal) and is able to cover large areas in a small amount of time. However, there are some limitations that should be considered. Firstly, we are not able to obtain at this stage, species-specific density estimates in these areas due to relatively high species diversity and patterns of co-occurrence on reefs. Furthermore, cryptic species cannot be easily detected due to the presence of an acoustic dead-zone next to the bottom that is larger in areas with high topographic complexity. Finally, because of the high variability that characterizes the crepuscular phases over short time periods, acoustic techniques were not always able to clearly capture the transitions due to limits on vessel speed over survey tracks. For all of these reasons, it is crucial to use additional observational tools, such as SCUBA diving, that are well suited to describe the species community, fine scale distribution and behavior patterns.

Spatial indices were able to capture changes of distribution patterns of predators and prey across space and time and to identify an overall opposite trend between the two groups. The use of the spatial indices was a very valuable tool and allowed us to easily summarize the differences between predators and prey even at these fine temporal and spatial scales.

The outcomes of this study highlight the extreme variability that characterizes sub-tropical reefs in terms of species interactions and spatial dynamics and there is the need to better understand such interactions in order to develop effective management for these habitats and their inhabitants. The increasing use of Ecosystem Based Management (EBM) has brought more attention to understanding processes across spatial and temporal scales, in particular predator-prey systems [63,64,65]. This study provides an example of how different tools can be integrated to better observe and define these interactions. The data obtained here can be potentially used in ecosystem end-to-end models [66,67] that have been gaining attention in this past 10 years because of their ability to model a wide range of processes at different scales, and these models can be used as a strategic tool to evaluate resource management scenarios to the whole ecosystem. End-to-end models are not largely used in an operative way at this time because of their complexity and data requirements, but they likely will be in the near future in order to fulfill the increasing needs of the EBM.

Future efforts should focus on understanding other elements that are important to explain the dynamics of predator-prey interactions at the GRNMS and how these relate to the demography of both predator and prey populations. In particular, evaluating the bioenergetic trade-offs in foraging behavior of the planktivorous prey species, including diel variation in zooplankton behavior as it effects availability, would improve understanding of the response of these species to predation risk and the tradeoffs between increasing growth, survival, and movement patterns to optimize feeding. Additional non-static factors such as hydrodynamics and productivity should be included in species distribution models in order to gain a more complete understanding of the elements of habitat selection. Finally, studies focused on the role that these interactions play in subsidizing production of higher trophic level predators links local-scale behavior to conservation objectives.

## Supporting information

S1 Fig Example of echograms showing the diel variability of fish behaviour and distribution at station 01OUT. The distance between the vertical line is 250 m. The horizontal line is at 10 m depth. A—night; B—predawn; C—postdawn; D—day; E—predusk; F- postdusk.
(TIF)

S1 File Acoustic dataset used in this paper for the habitat modeling and spatial analysis. (XLSX)

S2 File Visual census dataset from SCUBA diving observations. (XLSX)
