# Supplementary figures and images for: Dynamics of predator-prey habitat use and behavioral interactions over diel periods at sub-tropical reefs
Source: PLoS One. 2019 Feb 6;14(2):e0211886. doi: 10.1371/journal.pone.0211886 (PMC6364942; doi:10.1371/journal.pone.0211886)

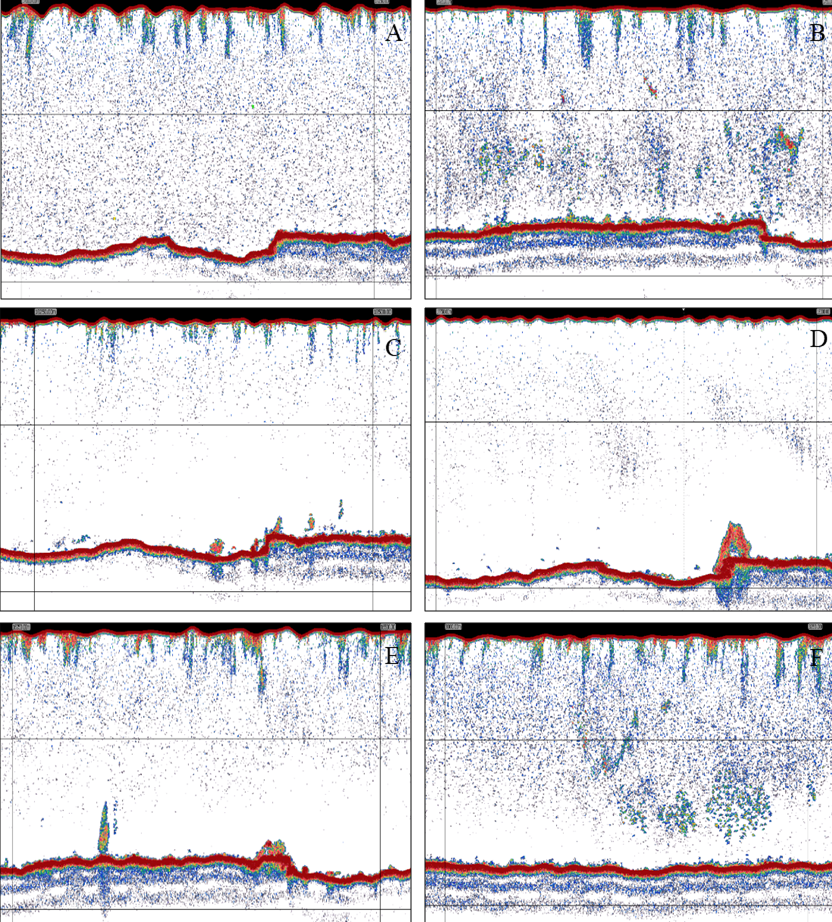

Supplement: S1 Fig — The distance between the vertical line is 250 m. The horizontal line is at 10 m depth. A—night; B—predawn; C—postdawn; D—day; E—predusk; F- postdusk. (TIF) [file pone.0211886.s001.tif]
